# Odiel River (SW Spain), a Singular Scenario Affected by Acid Mine Drainage (AMD): Graphical and Statistical Models to Assess Diatoms and Water Hydrogeochemistry Interactions

**DOI:** 10.3390/ijerph18168454

**Published:** 2021-08-10

**Authors:** José A. Grande, Ana Teresa Luís, Francisco Córdoba, Mercedes Leiva, José Miguel Dávila, Juan Carlos Fortes, María Santisteban, Eduardo Ferreira da Silva, Aguasanta Miguel Sarmiento

**Affiliations:** 1 Department of Water, Mining and Environment, Scientific and Technological Center of Huelva, University of Huelva, 21004 Huelva, Spain; grangil@uhu.es (J.A.G.); jmdavila@dimme.uhu.es (J.M.D.); jcfortes@uhu.es (J.C.F.); mariasantisteban@gmail.com (M.S.); aguasanta.miguel@dgeo.uhu.es (A.M.S.); 2 Sustainable Mining Engineering Research Group, Department of Mining, Mechanic, Energetic and Construction Engineering, Higher Technical School of Engineering, University of Huelva, 21007 Huelva, Spain; 3 GeoBioTec Research Unit—Department of Geosciences, University of Aveiro, Campus de Santiago, 3810-193 Aveiro, Portugal; eafsilva@ua.pt; 4 Department of Integrated Sciences, Faculty of Experimental Sciences, University of Huelva, 21071 Huelva, Spain; fcordoba@uhu.es (F.C.); mercedeslys@gmail.com (M.L.)

**Keywords:** Odiel River, acid mine drainage (AMD), hydro-geochemistry, Shannon–Wiener index, richness, diversity, factor analysis

## Abstract

The Odiel River (SW Spain) is one of the most cited rivers in the scientific literature due to its high pollution degree, generated by more than 80 sulphide mines’ (mostly unrestored) contamination in the Iberian Pyritic Belt (IPB), that have been exploited for more than 5000 years. Along the river and its tributaries, the physico-chemical parameters and diatoms, from 15 sampling points, were analyzed in the laboratory. Physico-chemical parameters, water chemical analysis, together with richness and Shannon–Wiener indexes were integrated in a matrix. An initial graphical treatment allowed the definition and proposal of a functioning system model, as well as the establishment of cause–effect relationships between pollution and its effects on biota. Then, the proposed model was statistically validated by factor analysis. For acidic pH waters, high values of Eh, TDS, sulphate, ∑REE and ∑Ficklin were found, while diatomologic indicators took low values. Thus, factor analysis was a very effective tool for graphical treatment validation as well as for pollution–biota interaction models’ formulation, governed by two factors: AMD processes and water balance suffered by the studied river. As a novelty, the cause–effect relationships between high barium concentration and low diversity and richness were demonstrated in the IPB, for the first time.

## 1. Introduction

The Odiel river basin is located in the Iberian Pyritic Belt (IPB) and its streams are affected by acid mine drainage (AMD), which originates from pyritic minerals’ contact with air and water. The pyrite comes from active and inactive mines (~88 in all IPB), exploited for more than 5000 years.

Acidic leachates from pyritic waste rock dams and (in lesser extent) coal mining, are the main acid mine drainage (AMD) producer focus.

When AMD is incorporated into rivers, notable changes in water hydro-geochemistry and biota are observed. The latter suffers alterations as a consequence of acidity and sulphate increase. AMD results from sulphides’ oxidation, which incorporates hydrogenions, sulphate, metals and metalloids into the aquatic environment, allowing fixation, bioaccumulation and biomagnification of pollutants in the water food chain. These processes are responsible for different pathological effects in biota, becoming lethal and always conditioning the species dominance and abundance, depending naturally on the pollution degree as well as on the adaptation capacity of each organism [1].

AMD is a global problem that affects the five continents and a standard solution to solve its effects has not yet been found. It is not only an economic issue for mining companies, that in Europe and USA are forced to extract and transform minerals with a “zero discharge” policy; it is also an ecological and social issue, as many areas, worldwide, are affected by mining waste from mines already closed, which predate the existence of rivers’ environmental discharge regulations [2].

In natural waters, the most important sources of acidity increase are: mine drainages, industrial discharges and acid rain. This water can have different acidification degrees: if it is slightly acidic, the alterations in the biota are low; however, increases in acidification also tend to make species disappear. For pH below 4.0, neither vertebrates nor most of the invertebrates or the autotrophic organisms are found. Some plants are capable of growing and reproducing in these polluted media and something similar occurs with animals living in rivers, ponds and lakes [3]. Sulphate pollution is normally associated with loss of water use, but this does not represent a serious problem for aquatic organisms, except for stenohaline [4]. The undesirable effects that high sulphate concentrations may have on water from mining rivers are masked by those created by acidity and heavy metals [1].

The hydrogeochemical characterization of inland water and the identification of aquatic organisms are indispensable tools to evaluate the water pollution degree as well as its adaptation capacity, and finally, to propose working lines for the use of some organisms as bioremediation/bioindicator agents [5,6].

Benthic algae are the main primary producers in aquatic ecosystems, since they are the first autotrophs of the food chain that feed the main primary consumers, especially invertebrates. As they are attached to a substrate, they can act as environmental indicators for physical, chemical or biological changes. Within the benthic algae are the diatoms, a bioindicator group recommended by the Water Framework Directive to assess water quality, given the following capacities [7,8]: (a) they can grow quickly and live in different conditions; (b) they are easy to collect; (c) they are sensitive to environmental changes.

Many studies on metal polluted rivers have shown that diatoms respond to environmental degradation not only at the community level through shifts in dominant taxa, diversity and richness patterns but also by morphological alterations of their cell walls (frustule), leading to cell size reduction [9,10]. Research on acidophilic and acid-tolerant algae showed that algae are tolerant to metals, having mechanisms to avoid toxic effects [11,12].

In this paper, richness (n of species) and diversity (Shannon–Wiener Index) were the diatomological parameters taken into account to define the ecological status of each sampling point in order to compare with AMD pollution degree, translated into acidic pH and high metal and sulphate concentrations.

Richness is the number of diatom species in a dataset or in a sample. For example, species richness of a dataset is the number of different species in the corresponding species list. Because richness does not take the species abundance into account, it is not the same thing as diversity, which does take abundance into account, as is shown in the Shannon–Wiener (diversity) index (Shannon 1948) formula:

(1) $$H`=-\overset{s}{\underset{i=1}{\sum }}\frac{n_{i}}{N}\log_{2}\frac{n_{i}}{N}$$

where: *s*—number of species; *n_i_*—the number of specimens of *i* species; *N*—total number of specimens.

The objective of this study is the determination of richness and diversity in the Odiel River Basin and sub-basins to establish cause–effect relationships between these two diatomological parameters and the hydrogeochemical variables of the watershed, that together will define water’s AMD pollution degree in response to the mining contaminants present in the surroundings.

## 2. Location Setting

**Figure 1:**
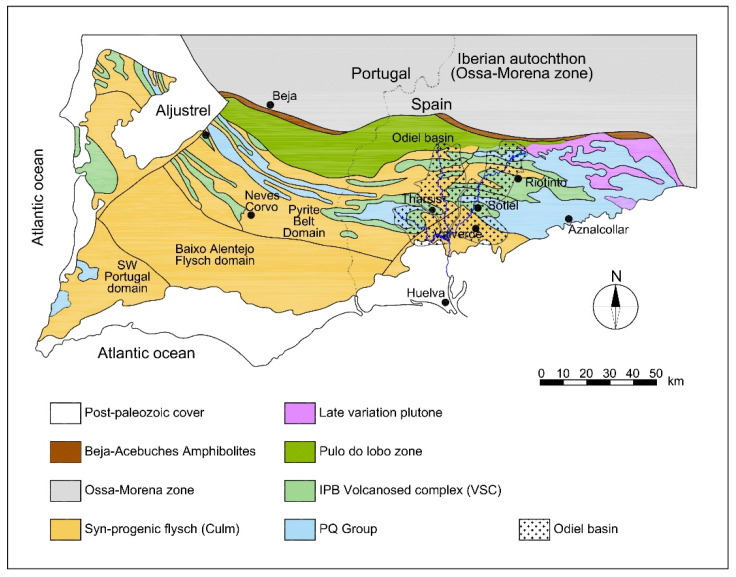
Geological map of the IPB with Odiel Basin marked.

**Figure 2:**
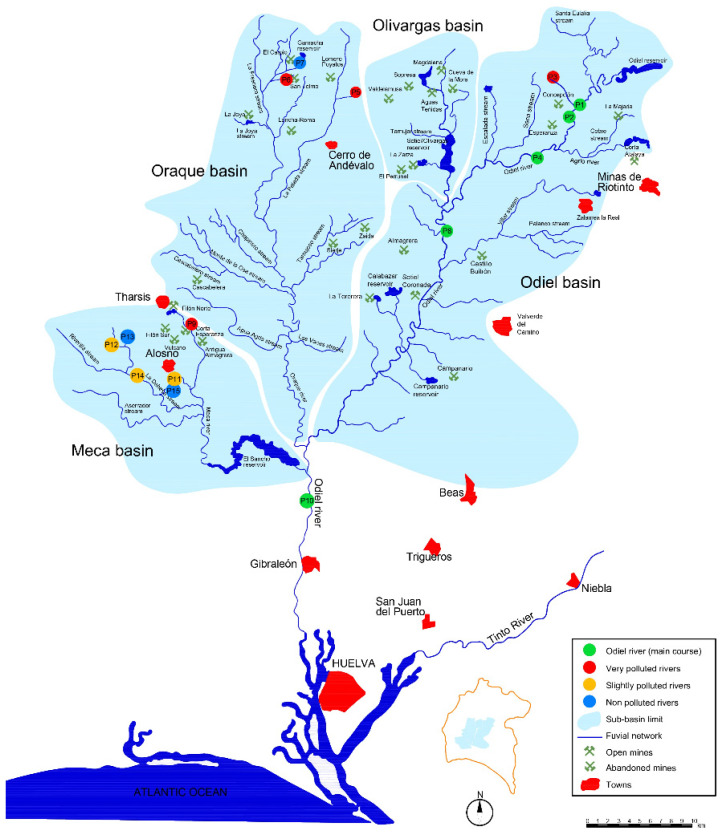
Map of the fluvial network with the sampling points’ location. The main channel corresponds to points 1, 2, 8, 10 and appears in green. Point 1 corresponds to Odiel before being contaminated and 10, the last one, before the tidal influence; the remaining points 2, 4, 8 correspond to downstream points. The rest of the sampling points are not in the same stream. They go from zero contamination degree: regional background = point 7 and points 13 and 15, also unaffected sites. The remaining points with color code orange and red were colored regarding their increasing contamination degree, slightly to very polluted sites, respectively.

The Iberian Pyritic Belt (IPB) extending from Lousal (Portugal) to Aznalcóllar (Seville) is one of the largest metallogenetic provinces of polymetallic sulphides (Figure 1), with 88 active and inactive mines (24 inactive and 6 active in the 4 basins of Figure 2), without preventive or corrective measures because the latter were exploited, before the environmental protection laws came into force, leaving more than 4000 ha of waste rock, open pit lakes and other mining facilities that continue to pollute rivers even after their abandonment [1], becoming an extraordinary scenario because of its extension and duration time: extractive activity with 5000 years of historical mining [13].

### 2.1. Hydrological and Mining Framework

The Iberian Pyritic Belt is north to south crossed by the Tinto and Odiel Rivers. The Odiel River (Figure 2) is longer (140 km) and has a catchment area [14] of 2069 km^2^. Both are affected by the presence of numerous mining operations in their catchment area. The Odiel River has four main sub-basins corresponding to Meca, Oraque, Olivargas and Odiel Rivers [15]. The Odiel and Tinto Rivers receive AMD contaminated inputs and were classified, in the past, as “Industrial Rivers” by the assigned environmental agency [16,17]. Thus, they were receptors of all kinds of non-treated effluents coming from the mines. The final result is a river network with extraordinarily high concentrations of dissolved metals and sulphates, with very low pH values. Average sulphate concentrations in the generator medium, close to mines, are above 20,000 mg/L, sometimes exceeding 100,000 mg/L, depending on rainfall. For Cu and Zn these values are situated between 300 and 500 mg/L, while Fe exceeds 6000 mg/L.

Many works have been carried out previously on the hydrogeochemical characterization of river basins at the IPB [13,18,19,20,21,22,23,24,25,26,27,28,29,30,31,32,33,34,35,36,37,38,39,40,41,42,43,44,45,46]. However, the works found on biota–hydrochemical relationships are very scarce. In this study field, the works [47,48,49,50] should be highlighted.

AMD pollution originates from when a sulphide mineral comes into contact with oxygen and atmospheric humidity [23,26,38,51,52]. On the mineral surface, a complex mechanism begins with the oxidation of other insoluble sulphides which are transformed into sulphates, with sulphuric acid production. This process is very slow, between 1.08 × 10^−15^ and 1.8 × 10^−14^ mol/cm^2^ s^−1^. Its speed is increased by a hundred times due to the presence of ferric ion [53] and catalytic bacteria [54]. Finally, at the same time as pyrite oxidation, secondary reactions take place between the previous reaction products and the minerals present in the rocks [55], obtaining a set of soluble contaminants deposited in the mineral, which are later dissolved and dragged by rainwater or runoff, producing a polluted and liquid flow, transporting acidity, sulphates and heavy metals to the water courses [56].

The phenomenon of sulphide oxidation is a natural process, but the speed is increased by the mining works, which allow a distinction between AMD natural geochemical process and anthropogenic origin AMD, caused by the massive amounts of ore that came to surface (in contact with oxygen) and by the increase in the contact surface due to mining activities and the consequent decrease in materials’ granulometry [57].

AMD effects are treated by mining companies and responsible agencies for land use planning. Effects once they appear are impossible to reverse, despite all current technology, because of the high costs associated; thus, they can remain for centuries [57].

### 2.2. Climatic Framework

Climatic conditions, and especially rainfall, are the most important external triggers and controlling factors evaluating the type and degree of mining contamination in any area. At the same time, climatic factors condition the survival capacity of the present species.

With slight variations along the Odiel River (as a function of latitude and altitude), the scenario under study is considered to be a semi-arid Mediterranean climate, with an annual rainfall between 650 and 750 mm/year condensed between November and March, with a discharge up to 70 mm/day and minimum evapotranspiration (<1 mm/day) [57]. The driest months coincide with July and August, with evapotranspiration up to 10 mm/day, although local storm events may occur during these months. This implies that the acid drainage contribution varies throughout the year and, consequently, the presence of biota in the waters. Annual average temperatures are in the order of 18 °C. In winter, they oscillate between 8 and 10 °C. In summer they are around 25–26 °C, with the maximum exceeding 40 °C. Very few days per year is the temperature below 0 °C; thus, the Odiel River never freezes.

It is worth noting the average value of evaporation potential (ETP), which is around 900 mm/year compared to the annual rainfall average, which means this is an area with a negative hydric balance and a higher ETP than the rainfall. This fact contributes to the existence of seasonal rivers that only transport water during the rainy season and sometimes after them, as a result of the waste rock’s “sponging”.

## 3. Materials and Methods

### 3.1. Sampling

To achieve the objectives, water samples were taken to be hydro-geochemically and biologically characterized. The sampling took place in February 2019 (beginning of spring in this type of climate), after the rainy season, when all of the described basin and sub-basins carry water. Fifteen representative sampling points were chosen to cover a wide range of pH, which are georeferenced in Figure 2. Water and diatom samples were collected both in mine-influenced (with a clear AMD contamination) and non-mine-influenced sites (four representative background points). The ephemerality of many of these streams did not allow a seasonal sampling per hydrological year, because, as said above, this region is subjected to a negative hydric balance (rain < ETP). Some watercourses carry water only in the rainy season and, in the case of streams coming from waste rock dams, a few more weeks in response to the water accumulated in waste rock dams. For diatoms, spring is the best moment to collect, because the communities increase diversity and abundance [58].

### 3.2. Field Measurements

pH, temperature, electrical conductivity (EC) and total dissolved solids (TDS) were measured in situ using a CrisonMM40 portable multimeter. Redox potential (Eh) was also measured in the field using a Hanna portable instrument. In each point, a water sample was taken and filtered immediately with 0.45 μm Millipore filters. Finally, suprapur nitric acid 2% was added to the water sample until it reached pH < 2 to fix the metals during their transport to the laboratory.

Sampling, treatment and identification of diatoms followed the Water Framework Directive (2006/118/EC) [59] guidelines. Sampling of diatom assemblages was carried out in the same 15 sites (Figure 2). Epipsammic diatom samples were collected by sucking the top layer of the sediments with a syringe. Following the sampling protocol in [60], pools of stagnant water and shaded sites were avoided. Two samples were taken, one kept alive and the other preserved in a formalin solution (5% final concentration).

### 3.3. Water Chemical Analysis

Water samples were analyzed by ICP-MS using a 7700 Agilent for the elements: As, Ba, Cd, Cr, Co, Cu, Mg, Mn, Mo, Ni, Pb, Sr, Ti, V, Zn and REE (lanthanides and actinides), and ICP-OES 5110 Agilent for the elements: Fe, Na, K, Ca, Si, P, S. Detection limit (LOD) was less than 0.5 µg/L for Rare Earths and less than 0.1 mg/L for the rest of the analyzed elements. International certified reference materials USGS GXR-1, GXR-2, GXR-4, and GXR-6 were analyzed at the beginning and end of each batch of samples. Internal control standards (Rh) were analyzed every ten samples and a duplicate was run for every ten samples. Reference standards were within 10% of certified values. The different solutions were prepared with Milli-Q (18.2 MΩ, 25 °C) water (Millipore, Bedford, MA, USA). The precision of all analyses was always within 5%. All reagents were of analytical grade or of Suprapure quality (Merck, Darmstadt, Germany). Stock standard solutions were Merck Certificate AA standard (Merck). Sulphates were determined by ion chromatography. All the analyses were carried out at the Central Research Services of University of Huelva. The services implemented a Quality and Environment system under the ISO 9001 and 14,000 standards, within the framework of compliance with these standards.

### 3.4. Diatom Analysis

Live diatom samples were examined in a light microscope to exclude the possibility of having dead diatoms and avoid abundance errors. From the other set of samples (preserved with formalin), an aliquot (after cleaning off the formalin) was treated following UNE-EN14407:2015 with hydrogen peroxide (H_2_O_2_ 30%) and heat was applied to accelerate the reaction, followed by three centrifugations (1500 rpm) to wash off the excess of H_2_O_2_ (with deionised water) in order to obtain a suspension of clean frustules. Permanent slides were mounted using the high refractive index (1.74) medium Naphrax^®^ (Brunel Microscopes Ltd., Chippenham, UK) (N.A. 1.32). Diatoms were identified (taxonomy was based on [60,61,62]) and semi-quantified (400 valves per sample) under a light microscope (Leitz Biomed 20 EB) using a 100X objective (N.A. 1.32) to determine relative abundances of species. Abundance and total number of species were used in the diatom assemblage diversity calculation through Shannon–Wiener index, using OMNIDIA (vs. 5.1), which is defined by the formula described in [63]:

(2) $$H`=-\overset{s}{\underset{i=1}{\sum }}\frac{n_{i}}{N}\log_{2}\frac{n_{i}}{N}$$

where *s*—number of species; *n_i_*—the number of specimens of *i* species; *N*—total number of specimens.

Richness was calculated as the number of different diatom species identified in each sample.

Diatoms’ preparation and identification were carried out at the biology lab of the Faculty of Experimental Sciences, University of Huelva. When taxonomical doubts were found, the clarification was carried out with the help of the Biology Department from the University of Aveiro, Portugal (GeoBioTec Investigation Unit).

### 3.5. Statistical Analysis

The field and chemical parameters, together with the different indices considered here, were integrated into a matrix for further processing. In a first approximation, given the large dataset obtained, the REEs were plotted as a summatory, as well as the most representative metals (same metals set considered in [64]).

To facilitate the comprehension of the water–metal–diatoms interaction, as well as the spatial evolution along the river, the standardization of all values was carried out between 1 and 10, as the variables showed, in some cases, extremely high values with respect to others. A pre-analysis of the physico-chemical parameters was conducted, integrating them in X–Y graphs which were then validated by a factor analysis (software package Statgraphics Centurion XVI).

## 4. Results and Discussion

### 4.1. Grouping of Sites

The physico-chemical data for each sampling point allowed the classification of sites according to a double criterion: the location and the AMD pollution degree, taking the pH as the main indicator of AMD pollution [1]. Thus, four different groups are presented in Figure 2:

1. Group 1 (Green): P1, P2, P4, P8 and 10, located in the Odiel riverbed.
2. Group 2 (Red): P3, P5, P6 and PP9 located in highly contaminated tributaries (pH < 3).
3. Group 3 (Orange): P11, P12 and P14, slightly affected by AMD (pH ~ 5), located in the Odiel River tributaries.
4. Group 4 (Blue): P7, P13 and P15, uncontaminated (pH ~ 7).

### 4.2. Graphical Treatment

**Figure 3:**
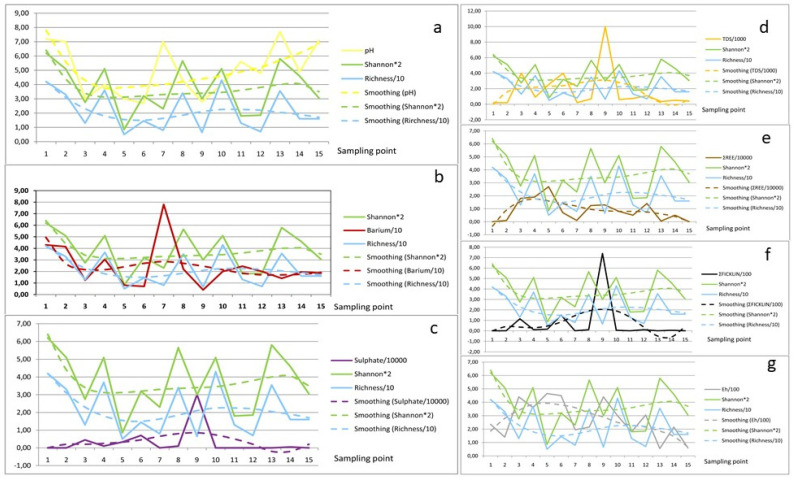
Spatial evolution of physico-chemical parameters: pH (**a**), barium (**b**), sulphate (**c**), TDS (**d**), ∑REE (**e**), ∑Ficklin (**f**) and Eh (**g**) vs. Shannon–Wiener index and richness.

Appendix A shows the values corresponding to the parameters of each sampling point. Note that their absolute value was standardized between 0 and 10 by means of multiplication/division factors for a better graphical visualization of the different physico-chemical variables’ behavior (ordered) at the different sampling points (abscissae), shown in Figure 3a–g.

Along the Odiel River and its tributaries (points 1 to 15), it is possible to observe the evolution of diversity (Shannon–Wiener Index) and richness, considered as dependent variables (biological) of the physico-chemical parameters.

From the total variables seven parameters were selected (Figure 3). The graphical treatment allows us to visualize the mass of data from a global point of view; thus, a hypothesis of river system functioning can be raised, i.e., a model of the cause–effect relationship between the physico-chemical parameters and the richness and diatoms’ diversity in each sampling point, which will be later validated by a statistical treatment from the same mass of data. Order 5 smoothing was carried out, in order to observe the evolution from a more distal perspective. Note how the trend lines of each study variable fit the proposed model much better, which is evidenced by a marked parallelism of all the trend lines. Point 15 requires a special mention, which will be explained later.

Figure 3a shows the relationship between pH and richness and diversity, making evident the direct relationship between them, as already described by different authors studying AMD-affected media [6,8,50]. It is evident, for all the variables, the higher the pH, the greater the diversity and consequently, the greater the species number. This phenomenon has been described for different scenarios. The highest diversity is found in point 1 that corresponds to the Odiel River before its contamination with Concepción Mine effluent, as the first contamination contributor.

In points 8 and 10 this relationship is somehow more discreet. In the case of point 10, this phenomenon is easy to interpret as it is a point located in the Odiel River estuary, in the tidal influence zone [14], where the pH suffers strong semi-daily nature oscillations with each tide, while diatom species are the response to the secular trend of the interannual pH value. The value introduced for this variable corresponds to a specific moment. There is high diversity, although the species with highest abundance are typical of acidic waters.

Point 8 does not have a simple explanation. Despite having a pH < 5, its diversity value is abnormally high. A high abundance of *Euglena* or filamentous algae type as *Klebsormidium* is observed in this area, immediately upstream of this sampling point. Furthermore, the presence of the Olivargas reservoir (clean water) could be a source of clean water, causing an increase in diatoms diversity and in a green algae bloom.

Following that premise, in points 7 and 15 with a pH above 7, a higher diversity value would be expected. Point 7 corresponds to a clean water reservoir of San Telmo Mining Group, which presents a high and positive anomaly in barium concentration, with respect to the other sampling points (Figure 3b). This relationship has been studied in Baja California by [65]. In that study a cause–effect relationship between the high barium concentration and the diatoms’ diversity was found. This finding was also described by other authors [66,67]. This phenomenon justifies the low richness and Shannon–Wiener (*H’*) values for such high pH. The presence of barium at this point is due to its use as a waterproofing layer for the dam wall used to supply drinking water to the San Telmo Mining Group.

In the case of point 15 the interpretation that: at high pH, there is low diatom richness and diversity, is simple. It is a small seasonal stream, with no mining inputs, that only transports water during the rainy season, leaving the riverbed dry for at least 8 months per year. This phenomenon suggests that diatom communities are sensitive to drought [68] and not all reappear year by year.

In addition, it is a point with low insolation due to the abundance of vegetation, which limits the growth of diatoms due to the absence of light. As would be expected, the lowest values of diversity (Shannon–Wiener) and number of species (richness) are found in the tributaries with a high degree of AMD pollution (3, 5, 6 and 9). Figure 3c compares the sulphate concentration with the biological indices. As can be seen, the sampling points with the highest AMD pollution degree (3, 5, 6 and 9) present the highest sulphate concentrations, a typical characteristic of watercourses affected by mining activities. [42,69,70]. Findings in [71] showed that a high concentration of sulphate significantly inhibits the species growth and photosynthesis, thus decreasing the species diversity and richness, as a response to such toxicity.

The AMD process begins with the oxidation of sulphides to give sulphates; the process involves, immediately, the release of hydrogenions and a consequent increase in water acidity. Abundant information on chemical reactions of AMD process is available in the scientific literature, and the most relevant studies are from [1,38,72]. This decrease in pH is simultaneous to the increase in sulphate concentration. Figure 3c shows a notable similarity with Figure 3a, in which the presence of sulphates in each sampling point is no more than the first “derivative” of the available concentration of primary oxidized sulphides, releasing hydrogenions and, obviously, decreasing the pH.

The relationship between the total dissolved solids (TDS) with diversity and richness is clearly similar to those from Figure 3c, as sulphate is the most abundant ion in aqueous AMD solutions, which, in this case, reached a maximum close to 10 g/L (Figure 3d). TDS in this type of environment is characterized by the numerical expression of the total of metals and metalloids dissolved in the aquatic environment, which have been incorporated as the acidity response to the water composition, contributing directly to the ecotoxicity. Salts are necessary for life; however, beyond certain concentration limits, diatoms become intolerant. In this way, it becomes clear that the most toxic waters show a lower diversity. This can be interpreted as the result of less tolerant species’ disappearance due to this extremely polluted environment.

With respect to the rare earth elements summatory (∑REE) (Figure 3e), there is a clear inverse relationship between REE concentration and diversity. Note that, at point 5, where diversity and species numbers falls, the highest REE value is found. Similarly, it is possible to observe that the lowest REE concentration (points 1, 2 and 13) corresponds to the highest level of diatoms’ richness and diversity.

Figure 3f compares the ∑Ficklin = [Cd] + [Co] + [Cu] + [Ni] + [Pb] + [Zn] and the species diversity and richness at each sampling point. This summatory is usually applied [10,64] to classify water according to the pH and concentrations of the mentioned metals.

As can be seen, the highest values for the ∑Ficklin are from the sites most affected by AMD. At these points, the Shannon–Wiener index and species richness decrease. This diagram indicates that, the greater the dissolved metal concentrations in water, the lesser the diatoms’ diversity and richness.

Figure 3g shows the inverse relationship between Eh and diatomological indices: the higher the Eh, the lower the species richness and diversity. It can be seen as the inverse relation between Eh and pH, which is consistent with data from [73] in systems affected by AMD.

**Figure 4:**
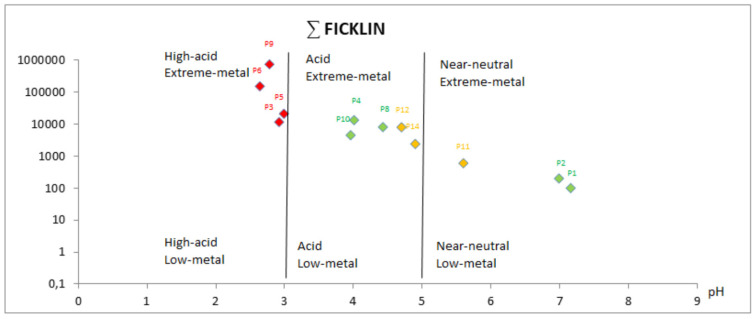
Ficklin diagram showing the sampling points’ grouping, accordingly to water type.

The Ficklin diagram (Figure 4) shows that in the most AMD contaminated points (3, 5, 6 and 9 in red), the metal concentrations are very high and the waters are extremely acidic, while the points with lower AMD contamination (11, 12 and 14 in orange) have a mean concentration of ∑Ficklin and a pH close to 5.

When studying the Odiel River sampling points in a strict sense (green colour), points 1 and 2 had very low metal concentrations and a neutral pH, as they are unaffected by AMD. Along the Odiel watercourse, as soon as we started to go down to the sea, the river receives more AMD contaminated effluents, which is why points 4, 8 and 10 have more acidic pH and higher metal concentrations.

Points 7, 13 and 15 were not represented in the diagram as they were unaffected by AMD and do not contain any of the Ficklin’s metals.

Thus, the Odiel River’s pH range goes from weakly alkaline water (carbonated) before contacting with AMD, to extremely acidic waters with very high concentrations of sulphates and metals.

### 4.3. Statistical Treatment

Table 1 shows the results of the statistical summary of the most significant variables, as a first approach of the statistical treatment, for a total of 15 sampling points. It is possible to observe that there is a high value dispersion around the central tendency (variation coefficient) for each variable, exceeding 266% in the case of ∑Ficklin or 236% in the case of sulphate. This fact is easily explained through the great differences of the sampling waters, from typically AMD-affected waters (pH values close to 2.6) to alkaline waters unaffected by mining contamination (pH close to 8). This leads to a great dispersion of the rest of the variables in response to acidity: both TDS and Eh, being closely associated with hydrochemistry, and the biological parameters related to diatoms’ presence in each point.

Something similar occurs with the range of the variables due to the different sampling scenarios, involving physico-chemically very different waters, with a pH range of 5 (subtracting minimum from the maximum). Remember that this is a variable expressed in a logarithmic scale and 5 pH units represent a high difference in the net acidity. Observing the sulphate, the range is extraordinarily high (30,689 mg/L), as a response to water’s dissolving capacity at very different pH. The same is observed for ∑REE and ∑Ficklin. As far as biological indicators are concerned, the richness range takes much higher values than Shannon–Wiener index; this is due to the Shannon–Wiener index concept, which itself is an indicator of “dispersion” and oscillates between 0 and 5 [63].

### 4.4. Factor Analysis

This technique has been widely used in the diagnosis of AMD pollution in waters, using physico-chemical parameters [52,74,75]. In this study and in addition to the physico-chemical parameters, two more variables were introduced: richness (number of different species) and diversity (Shannon–Wiener index), as input variables. Of note, and to facilitate the reading and interpretation of the factor analysis, the ∑Ficklin has been integrated into a single variable without disturbing the solvency of the model. In the same lines, the REE are also presented as a summatory, for the same reason.

**Figure 5:**
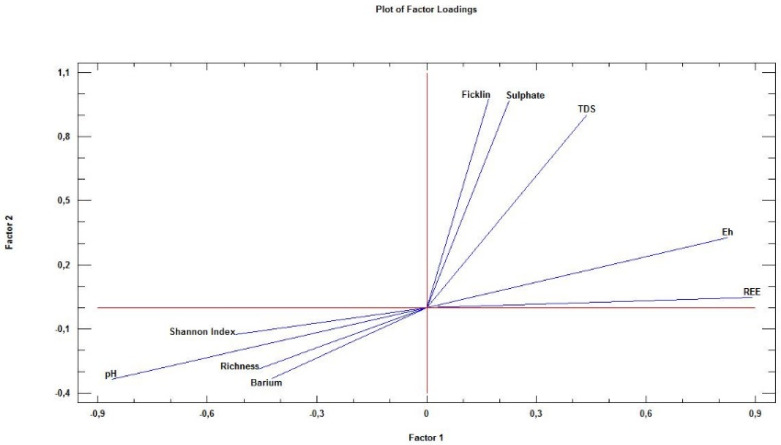
Plot of Factor 1 and Factor 2 correlation.

Figure 5 presents a factor analysis where two factors were obtained (defined previously) that explain most of the variables’ variability, achieving it as a whole. Only the first two factors account for up to 87.72% of the total variance (Table 2) and considering three factors, more than 98.65%. In summary, two factors (Factor 1, Factor 2) were defined, and not set in the field. They govern the mineral–water–biota interaction process and allow the definition of cause–effect relationships from all the information carried by the nine original variables, some of them already aggregated as a summatory (REE and Ficklin).

A first approximation to the model comes from the observation of a close proximity of Shannon, richness, barium and pH, all of them in the lower left quadrant; therefore, these have a negative correlation with Factors 1 and 2. In the opposite quadrant are the rest of the variables, clearly grouped in two groups. While ∑Ficklin and sulphate support a very high and positive correlation with Factor 2 and a slight correlation with Factor 1, ∑REE and Eh are highly and positively affected by Factor 1, and with a more discrete correlation with Factor 2. The TDS is in the middle, with high and positive correlation to both. The strict linearity between Eh and pH with a very high correlation coefficient already reported by several authors [73,76] should be highlighted. Therefore, in Figure 5 the two factors rotated by the varimax rotation method [77] suggest:

- Factor 1: Water balance. The most (and positively) affected variables are ∑REE and, at a short distance, Eh. The meaning of this is that the greater the water balance, the greater the values of these two variables. This phenomenon has already been described by [76]. At the opposite extreme, the variable pH is found; this fact is the response to the logarithmic character of pH and also because at lower pH, the water acidity is greater, and thus, the dissolution capacity is greater. Close to pH are the Shannon–Wiener and richness variables, as expected, as for extreme/low pH, extreme/low richness and diversity were found, a fact already described by [6,8,50,78,79]. It should be remembered that this region has a negative hydric balance, so in some of the sampling points, water will be found just in the rainy season. This fact has a significant impact on the survival capacities of different diatom species; thus, both richness and Shannon–Wiener are negatively affected by Factor 2. The same happens with barium, spatially associated with the previous ones in Figure 5, as it is an external element of the primary paragenesis of polymetallic sulphides and its presence is due to the anthropic activity when used as a waterproofing agent.
- Factor 2: AMD Processes. AMD processes are a determinant factor for the values taken by the variables under study. In fact, the sulphides’ oxidation into sulphates causes a release of hydrogenions and, therefore, the pH is directly and negatively affected by AMD processes. At the opposite extreme of Figure 5, three variables are found, most closely linked to AMD, ∑Ficklin, sulphates and TDS, because these variables are very highly and positively influenced by Factor 2. Factor 2 also exerts a positive influence, but much more discreet, on the dissolved REE and Eh. The reasons are different: while REE solubility product conditions its position in Factor 2 lower “weights”, as many of them are more soluble than heavy metals, the Eh is somehow more affected than the previous ones, but clearly lower than the typical AMD indicators—note how this variable appears diametrically opposite to pH, giving almost a specular line at the origin. This fact was already described by [73,76].

**Figure 6:**
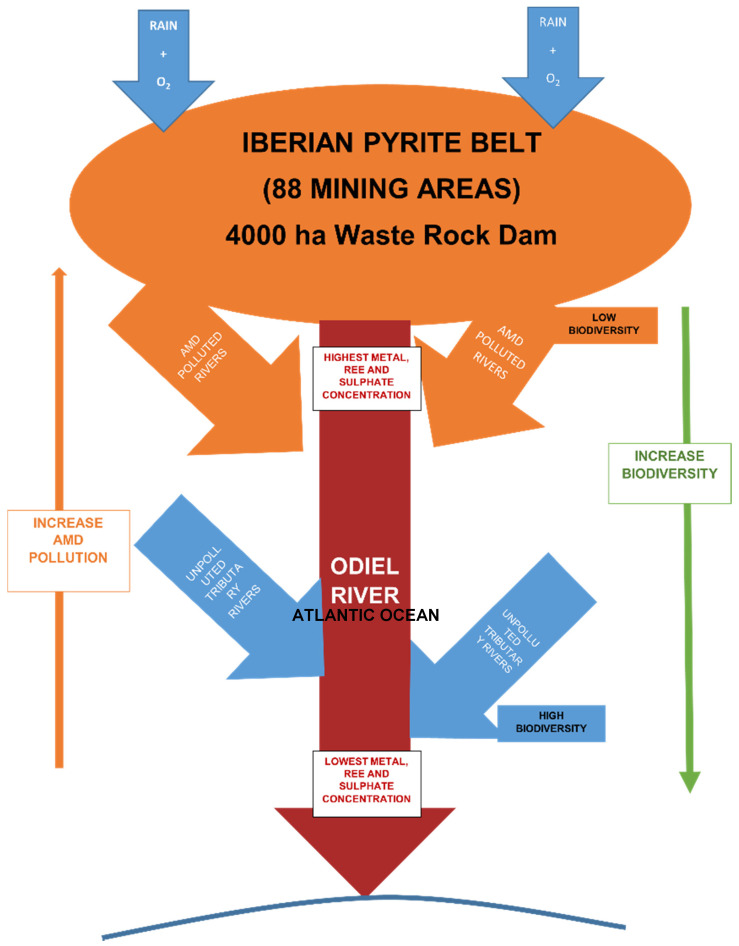
Graphical visualization of pollution degree and biodiversity evolution in the studied river.

The Figure 6 summarizes the chemistry and biodiversity evolution of waters in the studied watershed.

Notice that, when the proximity to the producing source increases, as well as the water pollution degree, the biodiversity decreases. This phenomenon is widely described in scientific literature regarding studied AMD media and was validated in this work, along the river path that crosses a whole metallogenetic province.

## 5. Conclusions

The present work was carried out in one of the most representative and polluted rivers wordwide, the Odiel River, which is part of a metallogenetic province (IPB) affected by AMD processes, coming from more than 80 sulphide mines. This study allowed the development of cause–effect relationships between diatom species’ diversity and richness and water hydrogeochemistry. For this purpose, graphical and statistical treatments of physical, chemical and biological data were carried out, concluding that sites of low AMD water show greater species diversity (Shannon–Wiener) and richness than those more affected by mining activities.

Specifically, by graphical treatment, it is evident that the most AMD-affected waters were from sites presenting more acidic pH and higher values of Eh, TDS, sulphate, ∑REE and ∑Ficklin (compared to those slighty or unaffected), while the biological parameters took lower values.

The factor analysis was carried out for the first time in this work for the water–biota–environment relationship. It is a very effective tool for graphical treatment validation (conducted with the same data matrix) and to formulate pollution–biota interaction models, governed by two factors: AMD processes and water balance suffered by the studied basin.

As a novelty, the cause–effect relationship between the high concentration of barium and the low diversity and species richness of diatoms, in this type of environment, is demonstrated. Despite that Ba is not in the mineral paragenesis, it has been incorporated into water due to its use as a waterproofing agent to dams of water storing for mechanical mineral treatment plants.

The results obtained coincide with those presented by other authors in different studied scenarios; however, in this work, the cause–effect relationships between physico-chemical parameters and diatoms were defined for the first time in a whole watershed and for different pH ranges. Thus, the ecological framework is conditioned by the referred thermo-pluviometric values and by the very variable hydrochemistry, along the sampling river.

## Figures and Tables

**Table 1:** Statistical summary of the 9 variables selected in the 15 sampling sites.

	pH	Eh (mV)	TDS (ppm)	Sulphate (ppm)	Barium (ppb)	∑Ficklin (ppb)	∑REE (ppb)	Shannon Index	Richness
Number points	15	15	15	15	15	15	15	15	15
Average	4.99	235.53	1787.07	3358.95	24.23	71.7	8768.29	1.88	21.8
Variation coefficient	36.22%	70.85%	146.89%	235.71%	78.09%	266.73%	94.94%	45.58%	64.95%
Minimum	2.64	16.0	155.0	18.0	3.58	0	113.39	0.44	5.0
Maxima	7.7	477.0	9950.0	30,707.0	78.2	741.6	2761.,1	3.11	43.0

**Table 2:** Factor analysis.

Factor Number	Eingenvalue	% of Variance	% of Cumulative Variance
1	4.88	67.85	67.85
2	1.43	19.87	87.72
3	0.79	1.93	98.65
4	0.08	1.10	99.76
5	0.02	0.24	100
6	0	0	100
7	0	0	100
8	0	0	100
9	0	0	100

## Data Availability

University of Huelva repository, http://hdl.handle.net/10272/7629 (accessed on 21 July 2021).

## Appendix A

**Table A1:** Values of the parameters of each sampling point. Their absolute value was standardized between 0 and 10 by means of multiplication/division factors.

Sampling Point	pH	Eh (mV) /100	TDS (mg/L) /1000	Sulphate (mg/L) /10,000	Barium (µg/L)/10	∑Ficklin (µg/L)/100	∑REE (µg/L) /10,000	Shannon-Wiener Index · 2	Richness /10
1	7.16	2.37	0.18	<0.01	4.40	<0.01	0.01	6.22	4.20
2	6.99	1.41	0.17	0.04	4.16	<0.01	0.04	5.14	3.40
3	2.92	4.36	4.08	0.51	1.22	1.23	1.77	2.68	1.30
4	4.01	3.65	0.82	0.09	3.07	0.13	1.87	5.14	3.70
5	2.99	4.77	2.71	0.35	0.79	0.21	2.76	0.88	0.50
6	2.64	4.52	4.12	0.80	0.65	1.53	0.78	3.24	1.50
7	6.90	1.95	0.16	0.01	7.82	<0.01	0.09	2.22	0.80
8	4.43	2.13	0.68	0.06	2.29	0.08	1.25	5.64	3.50
9	2.78	4.39	9.95	3.07	0.36	7.42	1.29	2.98	0.60
10	3.96	3.08	0.47	0.04	1.95	0.05	0.78	5.08	4.30
11	5.60	1.80	0.83	0.03	2.47	0.01	0.52	1.78	1.40
12	4.70	3.03	1.29	0.01	2.04	0.08	1.41	1.82	0.70
13	7.70	0.42	0.31	<0.01	1.32	<0.01	0.02	5.82	3.60
14	4.90	2.19	0.60	0.03	1.96	0.02	0.52	4.58	1.60
15	7.10	0.60	0.44	0.01	1.85	<0.01	0.03	3.06	1.60
